# A semi-supervised deep learning approach for predicting the functional effects of genomic non-coding variations

**DOI:** 10.1186/s12859-021-03999-8

**Published:** 2021-06-02

**Authors:** Hao Jia, Sung-Joon Park, Kenta Nakai

**Affiliations:** 1 Department of Computer Science, The University of Tokyo, 7-3-1 Hongo, Bunkyo-ku, Tokyo, 113-8656 Japan; 2 Human Genome Center, the Institute of Medical Science, The University of Tokyo, 4-6-1 Shirokanedai, Minato-ku, Tokyo, 108-8639 Japan

**Keywords:** Non-coding variants, Epigenome, Semi-supervised learning, Deep learning, Pseudo label

## Abstract

**Background:**

Understanding the functional effects of non-coding variants is important as they are often associated with gene-expression alteration and disease development. Over the past few years, many computational tools have been developed to predict their functional impact. However, the intrinsic difficulty in dealing with the scarcity of data leads to the necessity to further improve the algorithms. In this work, we propose a novel method, employing a semi-supervised deep-learning model with pseudo labels, which takes advantage of learning from both experimentally annotated and unannotated data.

**Results:**

We prepared known functional non-coding variants with histone marks, DNA accessibility, and sequence context in GM12878, HepG2, and K562 cell lines. Applying our method to the dataset demonstrated its outstanding performance, compared with that of existing tools. Our results also indicated that the semi-supervised model with pseudo labels achieves higher predictive performance than the supervised model without pseudo labels. Interestingly, a model trained with the data in a certain cell line is unlikely to succeed in other cell lines, which implies the cell-type-specific nature of the non-coding variants. Remarkably, we found that DNA accessibility significantly contributes to the functional consequence of variants, which suggests the importance of open chromatin conformation prior to establishing the interaction of non-coding variants with gene regulation.

**Conclusions:**

The semi-supervised deep learning model coupled with pseudo labeling has advantages in studying with limited datasets, which is not unusual in biology. Our study provides an effective approach in finding non-coding mutations potentially associated with various biological phenomena, including human diseases.

**Supplementary Information:**

The online version contains supplementary material available at 10.1186/s12859-021-03999-8.

## Background

It is well known that more than 95% of the human genome is non-coding DNA sequences that do not encode proteins [1]. Recently, many studies have found that these non-coding sequences play an indispensable role in biology. For example, genome-wide association studies have identified that the majority of variant loci (88%) associated with human diseases lie in non-coding regions and modulate gene regulation in a tissue- or cell-type-specific manner [2]. Some non-coding mutations introduce the gain and loss of functions of transcription factor binding sites [3], and the epigenomic modifications studied by large projects, such as Encyclopedia of DNA Elements (ENCODE) [4] and Roadmap Epigenomics [5], co-exist with the non-coding variants that are associated with diseases and traits.

For understanding the functional consequence of non-coding genetic variations, many researchers have utilized distinctive explanatory features and proposed computational tools. For example, FUN_LDA [6] is an unsupervised latent Dirichlet allocation model, and GenoSkyline [7] is trained by a two-component probabilistic mixture model. These two approaches calculate the prediction scores using histone modifications and DNase I hypersensitivity. Eigen [8] applies one unsupervised spectral learning method, and deltaSVMs [9] is a support vector machine (SVM) derived by the gkm-SVM classifier for the effective prediction of regulatory variants. CADD [10], a linear kernel SVM algorithm, and DANN [11], a deep learning model, utilize the same feature sets each other. DeepSEA [12], a deep learning-based framework, learns from sequence patterns in non-coding regions to predict allele-specific chromatin profile.

Over the past few years, the unsupervised machine learning and deep learning (DL) methods above mentioned have been successfully applied to this issue. However, these approaches rely on the input dataset and is refractory to the growth of its data scale [13]. In this context, many laborious experiments performed have ignored the fact that the number of non-coding variants experimentally validated is much fewer compared with millions of variants across the genome.

In this study, we propose a novel method employing a semi-supervised DL model with pseudo labels. To overcome the scarcity of available data, our method takes advantage of learning from both labeled and unlabeled data. Furthermore, we utilize epigenetic annotations and sequence features, which are observed from the genomic regions of non-coding variants to infer the important factors for the functional consequence.

## Results

### Overall structure of the proposed DL model

**Fig. 1:**
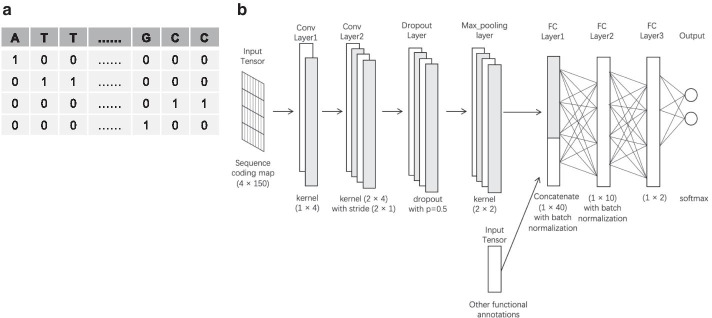
An overview of the deep neural network used in this study. **a** Sequence coding map of a 150-bp region centered by a non-coding variant locus. **b** Schematic representation of our network architecture. FC, fully connected

Recently, semi-supervised learning (SSL) has been extensively studied and has become more popular in various research fields [14–16]. In particular, the SSL coupled with pseudo labels, providing high-quality pseudo labels for unlabeled large-scale data during training, has been proven to allow the neural networks to make more confident predictions [17–19]. Taking its advantage, we developed an SSL model for analyzing genetic and epigenetic signatures in the 150-bp genomic regions where non-coding mutations occurred, as shown in Fig. 1.

As an input to our DL neural network, the nucleotides of a 150-bp region, centered by any non-coding variant locus, are represented by the binary vectors, as known as One-hot Encoding (Fig. 1a). Simultaneously, the 150-bp regions are scored by three scoring functions (i.e. Peak, Max, and Sum), measuring 10 histone enrichments and DNase sensitivity. The 10 different types of nucleotide compositions are also measured. These epigenetic and nucleotide composition features are concatenated with the output of the max-pooling function in our neural network structure (Fig. 1b).

### Predictive performance of our model and inference of impactful features

To test the feasibility of our approach, we downloaded the non-coding variant loci known in the human cell lines (GM12878, HepG2, and K562) [20]. Since these cell lines have been assayed extensively in ENCODE, we could access large-scale genomic and epigenomic data, which can be used to characterize the loci on a genomic scale.

**Fig. 2:**
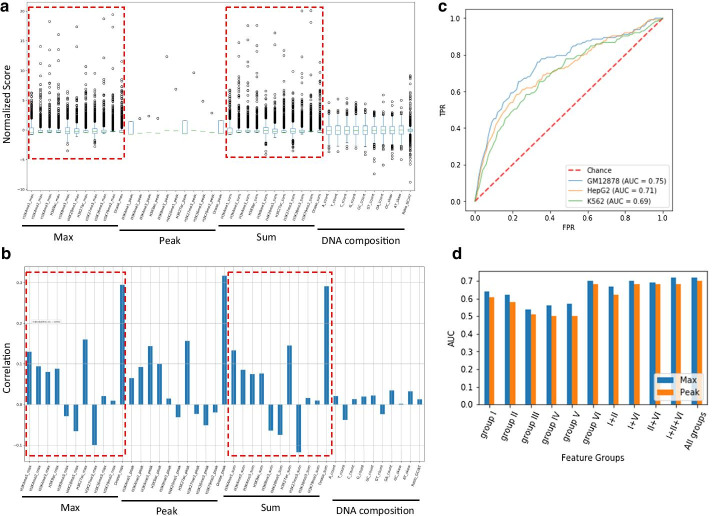
Feature distribution and the predictive performance of our deep learning model. **a** Plots showing the distribution of each score in the input feature map after preprocessing for K562. **b** Pearson correlation of each feature vector with the labels of non-coding variants in K562. **c** ROC curves showing the performance of our model. **d** AUC values showing the performance of our model with each of the grouped features in GM12878. *TPR* true positive rate, *FDR* false discovery rate, *AUC* area under the ROC (receiver operating characteristic) curve, *group I* histone marks on enhancers, *group II* histone marks on promoters, *group III* structural histone marks, *group IV* heterochromatin histone marks, *group V* histone marks on transcribed gene-body, *group VI* DNA accessibility assayed by DNase I sensitivity

First, we investigated the landscape of the input feature maps. In K562 cell line, the Max and Sum scores for the epigenetic marks showed broad ranges of distribution, and their patterns were greatly similar each other (Fig. 2a). These scores were similarly correlated with the distribution of non-coding variants, which was not observed in nucleotide composition features (Fig. 2b). Interestingly, the feature of DNase sensitivity was strongly correlated with the non-coding variants in all cases. Next, we assessed the performance of our model using the datasets shown in Table 1. As a result, although its performance in AUC reached to 0.75 in GM12878, no drastic differences were observed among cell lines (Fig. 2c).

**Table 1:** Experimentally labeled non-coding variant loci in three human cell lines

Cell lines	Positive	Negative	Total
Lymphoblastoid (GM12878)	683	2745	3428
Liver carcinoma (HepG2)	523	1432	1955
Erythroleukemia (K562)	340	1356	1696

*Positive* a locus affecting gene expression, *negative* a locus showing no effect

To examine what features contributed more to the performance, we grouped the epigenetic features into 6 functional categories; (I) Enhancer: H3K4me1 and H3K27ac, (II) Promoter: H3K4me2, H3K4me3 and H3K9ac, (III) Structural marks: H3K36me3 and H3K79me2, (IV) Heterochromatin: H3K9me3, H3K27me3, (V) Transcribed gene-body: H4K20me1, and (VI) DNA accessibility: DNase I sensitivity. As shown in Fig. 2d, the Max-score-based models with each of group I, group II, and group VI showed higher AUC values, consistent with the distribution of Pearson correlation in Fig. 2b. Remarkably, DNA accessibility (i.e. group VI) largely contributed to the performance. In contrast, nucleotide-based features, such as the GC-count, were less effective (Additional file 1: Fig. S1), in which the weak Pearson correlation in Fig. 2b may also explain this result.

Taken together, the epigenetic annotations, particularly DNA accessibility, are more explanatory for the presence of functional non-coding variants in K562. This result was also observed in GM12878 and HepG2 (Additional file 1: Fig. S2 and Fig. S3).

### Comparing with other models

**Fig. 3:**
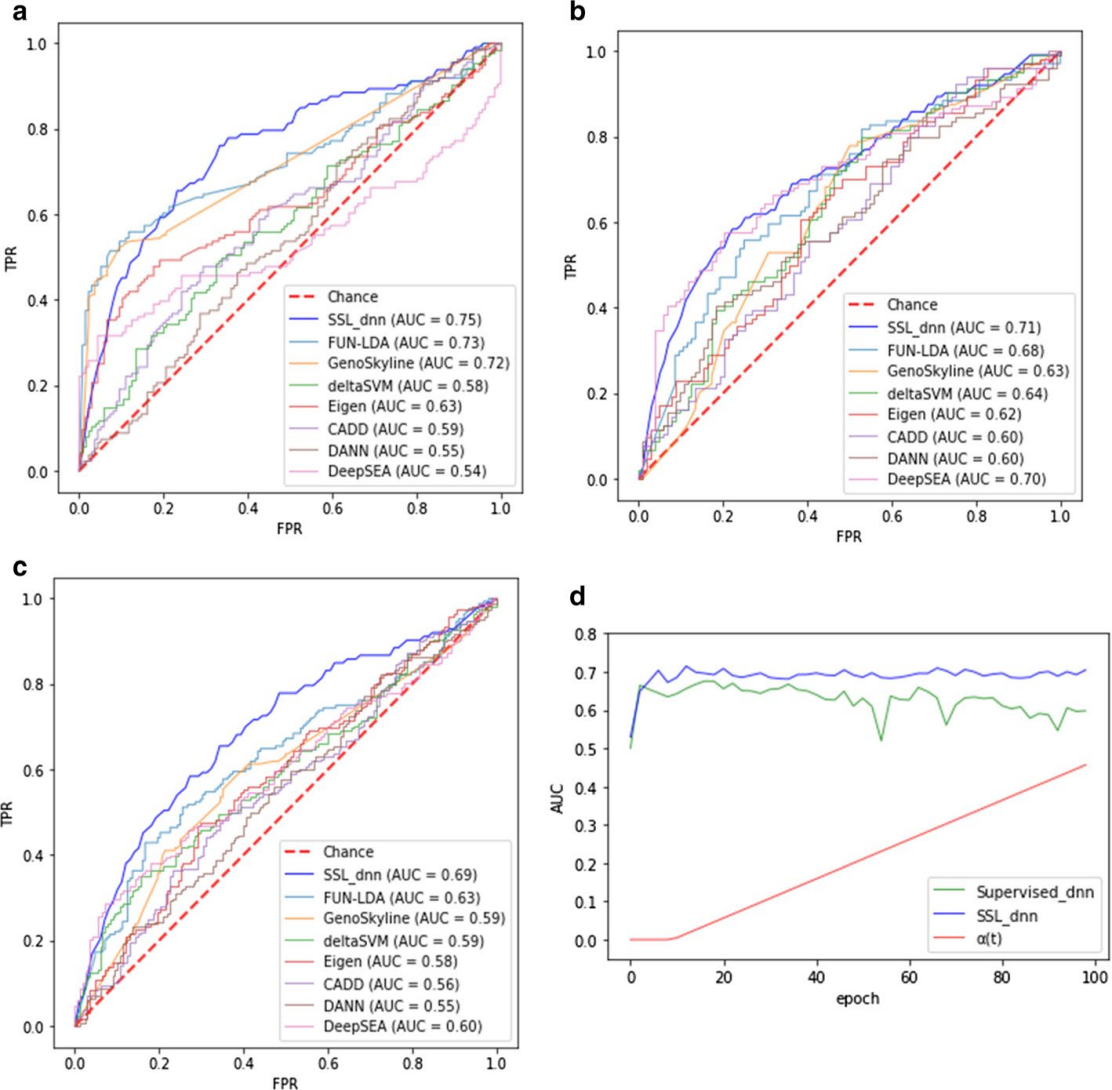
Comparing the performance of the proposed deep learning model with existing models and a supervised model. **a** ROC curve in GM12878 dataset. **b** ROC curve in HepG2 dataset. **c** ROC curve in K562 dataset. **d** AUC values showing the performance of the proposed model and a supervised model without pseudo labels in K562 dataset. AUC, area under the ROC (receiver operating characteristic) curve; Supervised_dnn, deep neural network; SSL_dnn, semi-supervised learning by dnn with pseudo labels; ${\upalpha }(\mathrm{t})$, a parameter in the loss function

We compared our semi-supervised learning by a deep neural network with pseudo labels (SSL_dnn) with seven existing unsupervised models; FUN-LDA [6], GenoSkyline [7], Eigen [8], deltaSVM [9], CADD [10], DANN [11], and DeepSEA [12]. Due to the technical difficulties of implementing these classifiers, we downloaded their prediction scores for each non-coding variant locus from the previous study [20] that performed the prediction with the classifiers and the three cell lines. By applying SSL_dnn to the same validation dataset, we could draw AUC curves and compare them. As a result, SSL_dnn exhibited higher AUC values; 0.75 in GM12878, 0.71 in HepG2, 0.69 in K562 (Fig. 3a–c).

Next, we compared SSL_dnn with a supervised deep neural network without pseudo labels. Their architectures and running parameters were exactly the same, but the supervised model considered only the supervised loss during the training process. As shown in Fig. 3d, although the classifiers showed similar growth trends for AUC values at the beginning, SSL_dnn gradually obtained better performance as the epoch increased in K562 cell line. It is expected that the two classifiers are practically the same algorithms at the beginning since the cross-entropy loss function in SSL_dnn composes only labeled loss [i.e. ${\upalpha }\left(\mathrm{t}\right)=0$]. When the dynamic schedule of ${\upalpha }(\mathrm{t})$ starts incorporating unlabeled loss in the cross-entropy loss function, the performance of SSL_dnn is outstanding, which suggests the impactful contribution of pseudo labels.

Consequently, we confirmed that the proposed model outperforms the current unsupervised models and supervised ones without pseudo labels in terms of AUC value, utilizing both experimentally confirmed labeled data and a large amount of unlabeled data.

### Predicting non-coding variants in specific cell lines

**Fig. 4:**
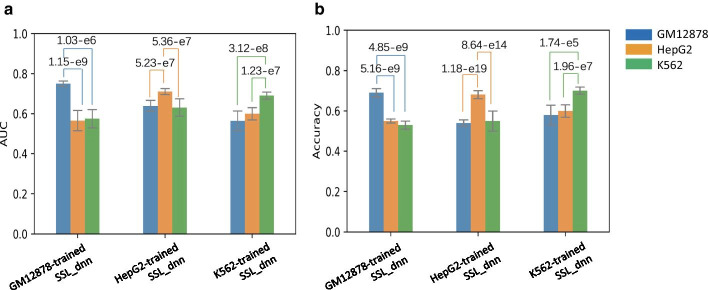
The performance of the proposed model trained with a certain cell-line dataset and evaluated with the validation datasets of other cell lines by AUC values (**a**) and Accuracy values (**b**). The bars represent the standard errors in fivefold cross validation and p-values were calculated by two-tailed *t*-test; AUC, area under the ROC (receiver operating characteristic) curve; SSL_dnn, semi-supervised learning by a deep neural network with pseudo labels

To investigate whether the nature of non-coding variants is cell-type specific or promiscuous, we trained SSL_dnn with the dataset of a certain cell line and predicted the validation datasets of other cell lines. Then, we evaluated its performance using AUC as well as Accuracy, the fraction of correctly predicted labels in total loci. As shown in Fig. 4, the models did not exhibit satisfactory predictive performance for the variant loci in other cell lines that are not used for the training. This result suggests that the non-coding variants are involved in cell-type specification, accompanied by different variant loci and distinctive histone modifications.

## Discussion

It is known that pseudo labeling helps to exploit the prediction of the DL model with ground truth for unlabeled data and also enables the DL to learn from the unlabeled data. This algorithmic uniqueness has given a new window to study various biological phenomena with smaller numbers of experimentally confirmed data and a large amount of relevant data unannotated. In this study, we developed a semi-supervised DL model with pseudo labels to predict the functional effects of non-coding variants.

We confirmed that the deep neural network exploits the pseudo labels assigned for unlabeled data and the labeled data updated with these pseudo labels during the training process. After setting up fair comparisons as possible, our approach was prominent compared to the existing unsupervised classifiers and the supervised classifier without using pseudo labels under the same setting. Of note, we used the imbalanced number of positive and negative data in the unlabeled datasets (Table 2). When adding the imbalanced unlabeled data to the training process, the performance of our model became higher than that of the supervised classifier (Fig. 3d). This result indicates that the imbalance of the training dataset may not negatively affect the performance of SSL, which requires further detailed studies.

**Table 2:** Training and validation datasets prepared from the loci in Table 1

Cell lines	Training		Validation (pos/neg)
	Labeled (pos/neg)	Unlabeled (pos/neg)	
Lymphoblastoid (GM12878)	550 (275/275)	2606 (272/2,334)	272 (136/136)
Liver carcinoma (HepG2)	450 (225/225)	1297 (197/1,103)	208 (104/104)
Erythroleukemia (K562)	350 (175/175)	1210 (97/1,113)	136 (168/168)

*pos* a positive locus affecting gene expression, *neg* a negative locus showing no effect

Through the investigation of impactful features for the prediction, we found that DNA accessibility reflecting open chromatin status [21] is the most indispensable feature (Fig. 2). This feature exhibited a relatively higher correlation with the distribution of functional non-coding variants. In contrast, the features based on nucleotide compositions were less effective. Importantly, our model trained with the dataset in a certain cell line is unlikely to succeed in predicting the variants in other cell lines. These results suggest that cell-type-specific epigenetic factors related to open chromatin conformation interplay with the functional non-coding variants.

We here employed the experimental annotations and epigenomic data in human cell lines, which allowed conducting the validation of our method and the characterization of the non-coding variations on a genomic scale. As future works, the extensive assessment with human disease samples and the incorporation of more comprehensive annotations require, which will give insights into how and why the non-coding variants are involved in diseases and traits.

## Conclusions

The semi-supervised deep learning model coupled with pseudo labeling has advantages in studying with limited datasets, which is not unusual in biology. Our study provided an effective approach to find non-coding mutations potentially associated with various biological phenomena including human diseases.

## Methods

### Preparing datasets

We downloaded the non-coding variant loci and their labels in GM12878, HepG2, and K562 cell lines from a previous study [20]: label 1 for positive loci affecting the gene regulation and label 0 for negative loci that are nothing to do with gene expression (Table 1). In addition, we downloaded the processed datasets of histone modifications and DNase I sensitivity from ENCODE. The histone ChIP-seq data included H3K4me1, H3K4me2, H3K4me3, H3K9ac, H3K9me3, H4K20me1, H3K27ac, H3K27me3, H3K36me3, and H3K79me2.

### Generating feature vectors

After checking the overlap of non-coding loci with the epigenetic marks, we generated three types of feature vectors: (1) Peak, 1 for a non-coding variant locus positioned within a peak region of an epigenetic mark, zero for others; (2) Max, the maximum enrichment score within a 150-bp region centered by a non-coding variant locus; (3) Sum, the sum of enrichment score for the 150-bp region. We calculated the nucleotide compositions of the 150-bp region: (4) Mononucleotide count, A_count (Adenine), T_count (Thymine), G_count (Guanine), and C_count (Cytosine); (5) Dinucleotide count, GC_count, GT_count, and GA_count; (6) Skew, AT_skew [$=(\mathrm{A}\_\mathrm{count}-\mathrm{ T}\_\mathrm{count})/\mathrm{AT}\_\mathrm{count}]$ and GC_skew [$=(\mathrm{G}\_\mathrm{count}-\mathrm{ C}\_\mathrm{count})/\mathrm{GC}\_\mathrm{count}];$ (7) Ratio of skew (= $\mathrm{GC}\_\mathrm{skew}/\mathrm{AT}\_\mathrm{skew}).$In addition, we encoded each base in the 150-bp region by adopting the one-hot encoding; [1,0,0,0] for A, [0,1,0,0] for T, [0,0,1,0] for C, and [0,0,0,1] for G.

### Implementing a deep learning (DL) model

Our DL model consists of two convolutional neural layers that deal with the code matrix with the shape of 150 (sequence length) × 4 (size of one hot coding approach). The output channel sizes in the convolutional layers are 2 and 4, respectively. The first convolutional neural layer uses a (1 × 4) convolutional filter with no padding for the information extraction from nucleotide vocabularies, while the second one applies a (2 × 4) filter and a (2 × 1) stride step. We employed the dropout function as the third layer. This function randomly assigns zero for some hidden units, making them be omitted during training, which contributes to minimize overfitting [22]. We used a max-pooling layer with the kernel size of (2 × 2), reserving the maximum values in windows and leaving a dense feature map with the size (4 × 1 × 72) to the next layer. We also used the ReLU (Rectifie Linear Units) function [23] as the activation method for each neural unit.

Our model included three fully-connected (FC) layers, which are also known as dense layers, with the sizes of 40, 10, and 2, respectively. The input to the first FC layer is generated by concatenating the output of the max pooling function with the additional feature map of the epigenetic and nucleotide composition features. We added the dropout function and the batch normalization function to the first and second FC layers, making non-linear transformations for the incoming data [24]. After the third FC layer, we applied the ReLU activation function. The final output layer consists of two neural units that correspond to the probability of two classifications.

### Implementing a semi-supervised DL model with pseudo labels

The concept of model training with pseudo labels for real unlabeled and large-scale data has been proved. As briefly, the prediction $\widehat{{\mathrm{y}}_{l}}$ of a deep neural network is given by

$$\widehat{{\mathrm{y}}_{l}} :=\mathrm{ argmax }{\mathrm{f}}_{\uptheta }{\left({\mathrm{x}}_{\mathrm{i}}\right)}_{\mathrm{j}} ,$$

where ${\mathrm{f}}_{\uptheta }$ is the function directly mapping the input space ${\mathrm{x}}_{\mathrm{i}}$ to confidence scores. The output is a two-dimensional vector for each input feature map. The network is trained by minimizing the cross-entropy loss $\mathrm{L}$ given by

$$\mathrm{L}= {\mathrm{L}}_{\mathrm{label}}+\mathrm{ \alpha }{\mathrm{L}}_{\mathrm{unlabel}} ,$$

where ${\upalpha }$ is a coefficient set by considering the tradeoff for labeled versus unlabeled conditions. The ${\upalpha }$ at the current batch $t$ is defined by the dynamic function [23]; 0 when $t<{T}_{1}$, $\frac{{t-T}_{1}}{{T}_{2}-{T}_{1}}$ when ${T}_{1}<t<{T}_{2}$, otherwise 1.

For training our deep neural network, we first divided the labelled dataset of a cell line into three parts: labeled and unlabeled datasets for training, and a validation dataset for testing (Table 2). In order to make the labeled dataset and validation dataset balanced, the remaining positive loci is much fewer than the negative loci in the unlabeled dataset. Using the training datasets, we performed the iterative training process by initializing with random parameters: the process with the labeled dataset was monitored by a supervised loss term, then the unlabeled dataset was predicted by the trained model. The class that had the maximum predicted probability in the two-dimensional output vector was selected as the “real” label to train the model. Then, the cross-entropy loss was calculated for optimizing the model. Of note, the number of unlabeled data decreases during the iteration as the unlabeled data with the most confident pseudo-labels is added to the labeled datasets to be used in the next epoch.

### Parameter setting

We used the stochastic gradient descent function [25] to update the parameters with the learning rate 0.03. We set the mini-batch sizes for the labeled and unlabeled training datasets and for the validation dataset to 16, 32, and 20, respectively. The threshold to select the confidential pseudo-labels was 0.95. We used ${T}_{1}=100, {T}_{2}=600$.

## Supplementary Information

**Additional file 1. Fig. S1**: AUC values showing the performance of our model with each of the grouped features in K562 in order to test the contribution of the 33 epigenetic annotations and context sequence that was used in this work. AUC, area under the ROC (receiver operating characteristic) curve; Nuc-based, nucleotide composition; Seq-based, sequence coding map. **Fig. S2**: Feature distribution of our deep learning model. (a) Plots showing the distribution of each score in the input feature map after preprocessing for GM12878. (**b**) Pearson correlation of each feature vector with the labels of non-coding variants in GM12878. **Fig. S3**: Feature distribution of our deep learning model. (**a**) Plots showing the distribution of each score in the input feature map after preprocessing for HepG2. (**b**) Pearson correlation of each feature vector with the labels of non-coding variants in HepG2.

## Data Availability

The histone ChIP-seq and DNase I datasets were downloaded from the ENCODE repository at https://www.encodeproject.org/: E116 (GM12878), E118 (HepG2), and E123 (K562). The processed results are available either as supplementary data or upon request.

## Abbreviations

AUC: Area under ROC curve
DL: Deep learning
Encode: Encyclopedia of DNA Elements
ReLU: Rectifie Linear Unit
ROC: Receiver operating characteristic
SSL_dnn: Semi-supervised learning by deep neural network with pseudo labels
SSL: Semi-supervised learning
SVM: Support vector machine
