# Supplementary figures and images for: A semi-supervised deep learning approach for predicting the functional effects of genomic non-coding variations
Source: BMC Bioinformatics. 2021 Jun 2;22(Suppl 6):128. doi: 10.1186/s12859-021-03999-8 (PMC8171027; doi:10.1186/s12859-021-03999-8)

Figure S1

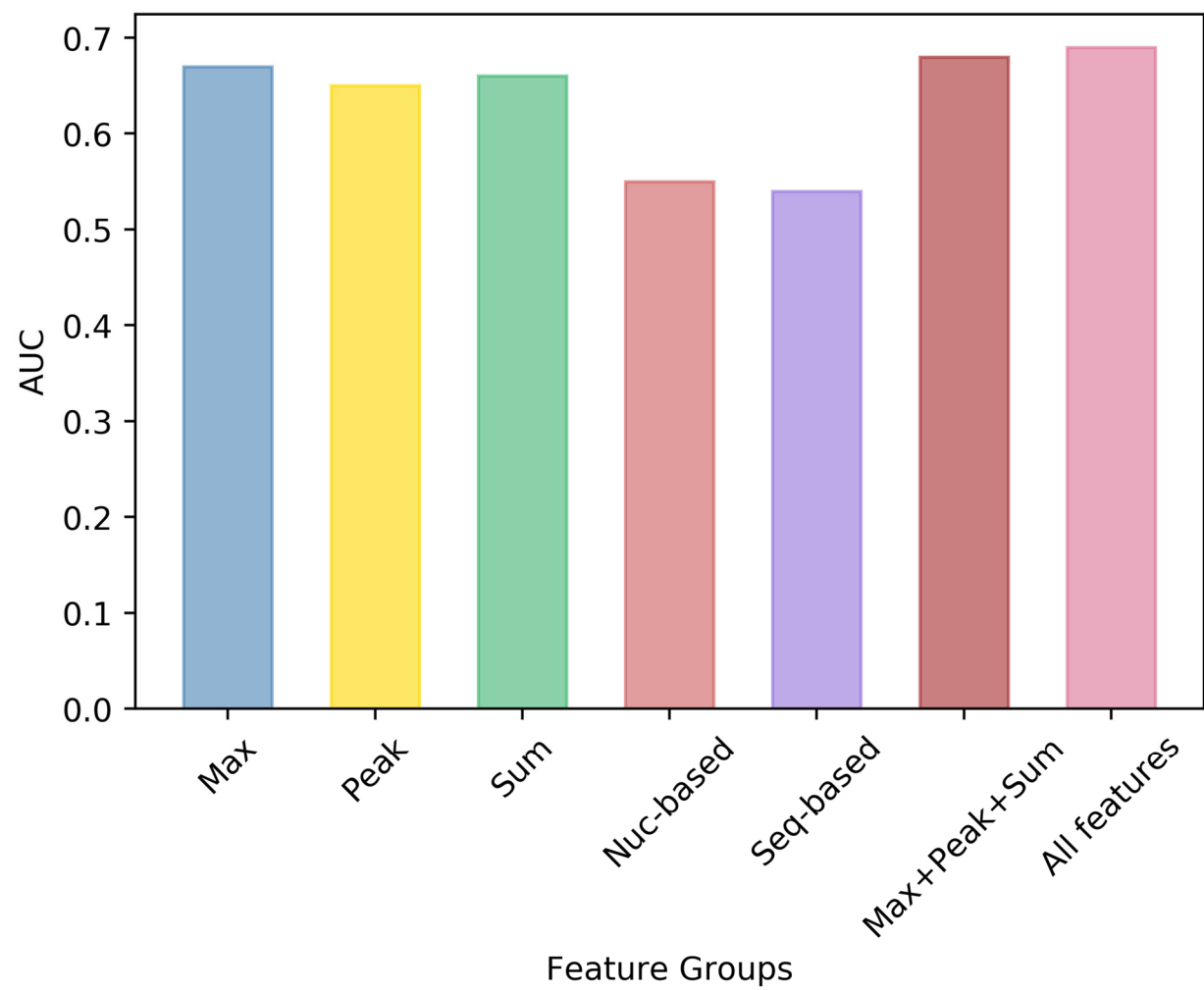

(a)

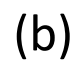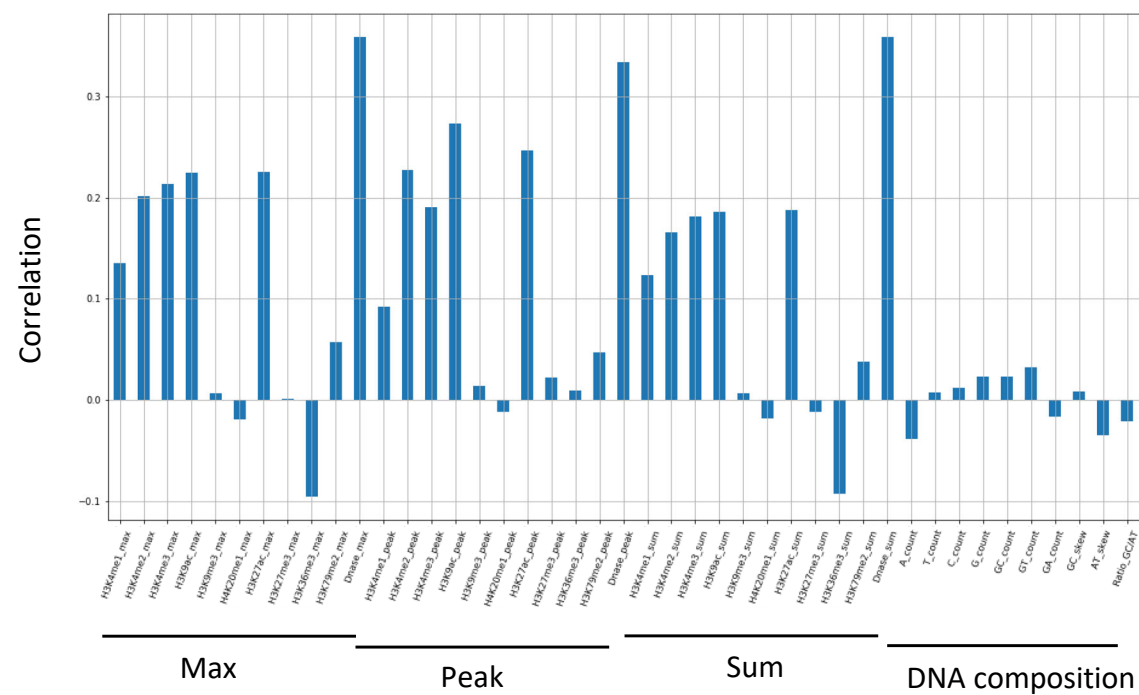

Figure S3

(a)

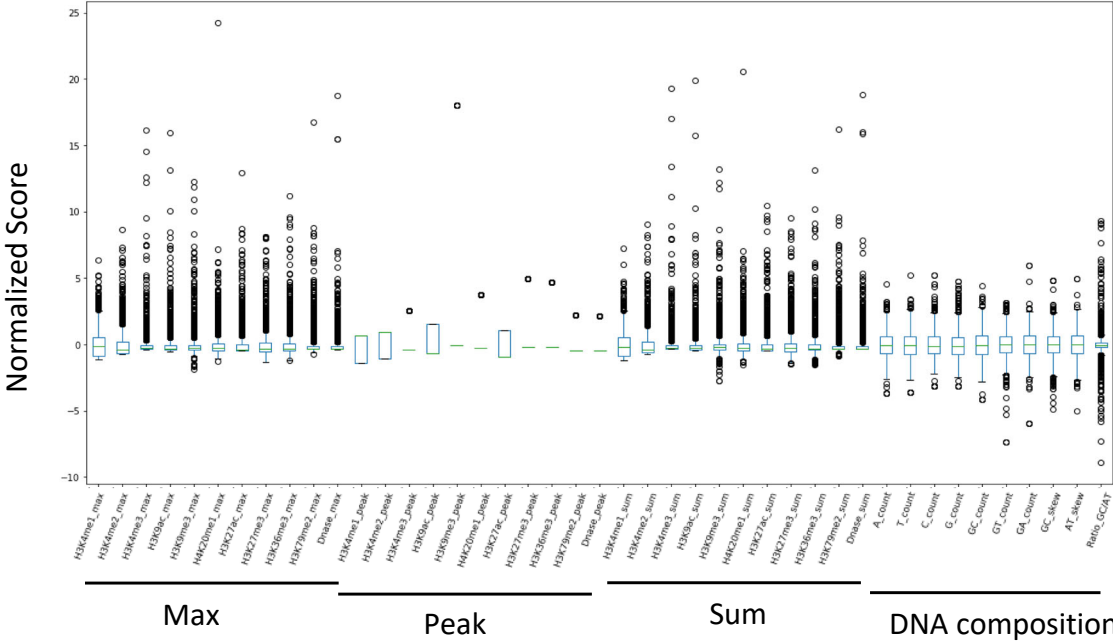

(b)

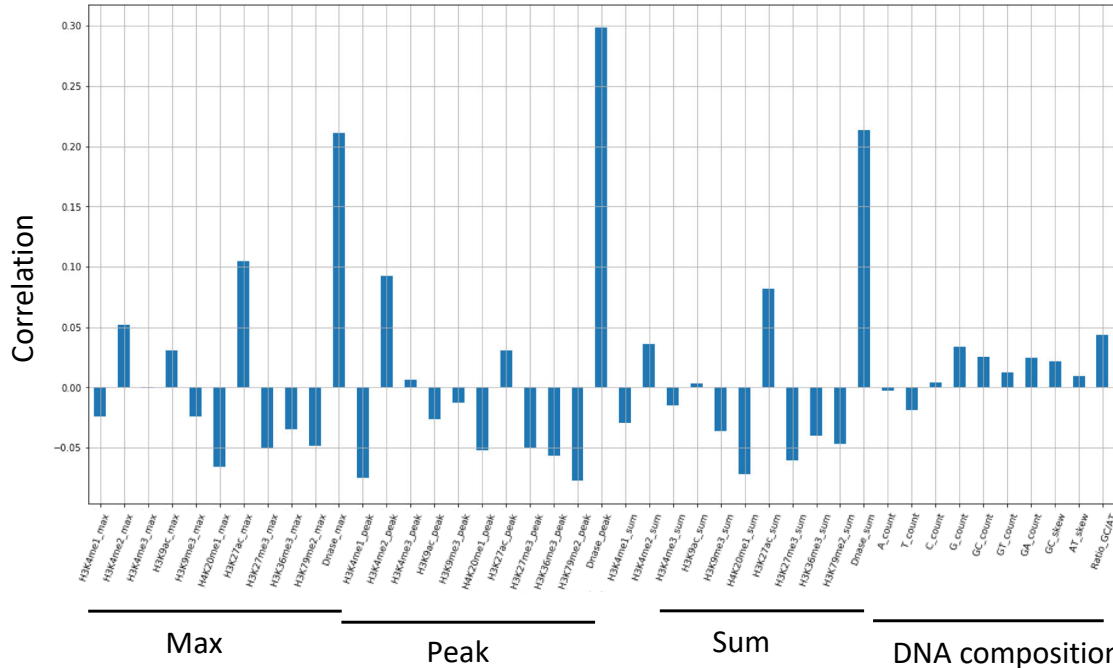

Supplement: Supplementary file 1 — Additional file 1. Fig. S1: AUC values showing the performance of our model with each of the grouped features in K562 in order to test the contribution of the 33 epigenetic annotations and context sequence that was used in this work. AUC, area under the ROC (receiver operating characteristic) curve; Nuc-based, nucleotide composition; Seq-based, sequence coding map. Fig. S2: Feature distribution of our deep learning model. (a) Plots showing the distribution of each score in the input feature map after preprocessing for GM12878. (b) Pearson correlation of each feature vector with the labels of non-coding variants in GM12878. Fig. S3: Feature distribution of our deep learning model. (a) Plots showing the distribution of each score in the input feature map after preprocessing for HepG2. (b) Pearson correlation of each feature vector with the labels of non-coding variants in HepG2. [file 12859_2021_3999_MOESM1_ESM.pdf]
